# Design and Fabrication of a Novel Corona-Shaped Metamaterial Biosensor for Cancer Cell Detection

**DOI:** 10.3390/mi14112114

**Published:** 2023-11-18

**Authors:** Nourelhouda Dadouche, Zinelabiddine Mezache, Junwu Tao, Enas Ali, Mohammad Alsharef, Abdullah Alwabli, Amar Jaffar, Abdullah Alzahrani, Achouak Berazguia

**Affiliations:** 1 Institute of Optics and Precision Mechanics, University of Ferhat Abbas Setif, Setif 19000, Algeria; mkh.nourelhouda@yahoo.com (N.D.); berazguiaachouak@gmail.com (A.B.); 2 LAPLACE, INP-ENSEEIHT, 2 Rue Camichel, 31071 Toulouse, France; tao@laplace.univ-tlse.fr; 3 University Toulouse III, 118 Route de Narbonne, CEDEX 9, 31062 Toulouse, France; 4 Faculty of Engineering and Technology, Future University in Egypt, New Cairo 11835, Egypt; enas.ali@fue.edu.eg; 5 Department of Electrical Engineering, College of Engineering, Taif University, P.O. Box 11099, Taif 21944, Saudi Arabia; m.alsharef@tu.edu.sa (M.A.); aatyah@tu.edu.sa (A.A.); 6 Department of Electrical Engineering, College of Engineering and Computing in Al-Qunfudhah, Umm Al-Qura University, Mecca 21955, Saudi Arabia; aswabli@uqu.edu.sa; 7 Computer and Network Engineering Department, College of Computing, Umm Al-Qura University, Mecca 21955, Saudi Arabia; ayjaafar@uqu.edu.sa

**Keywords:** metamaterial resonator, cancer, biosensor, network analyzer, corona form

## Abstract

The early detection and diagnosis of cancer presents significant challenges in today’s healthcare. So, this research, suggests an original experimental biosensor for cell cancer detection using a corona-shaped metamaterial resonator. This resonator is designed to detect cancer markers with high sensitivity, selectivity, and linearity properties. By exploiting the unique properties of the corona metamaterial structure in the GHz regime, the resonator provides enhanced interaction of electromagnetic waves and improved detection skills. Through careful experimental, simulation, and optimization studies, we accurately demonstrate the resonator’s ability to detect cancer. The proposed detection system is capable of real-time non-invasive cancer detection, allowing for rapid intervention and better patient outcomes. The sensitivity value was confirmed through simulation, estimated at 0.1825 GHz/RIU. The results of two different simulation methods are used: the simulation software CST Studio Suite (version 2017) based on the finite element method (FEM), and the simulation software ADS (version 2019) based on the equivalent circuit method, thereby increasing confidence in the convergence of simulation and measurement results. This work opens new avenues for developing advanced detection technologies in the field of oncology, and paves the way for more effective cancer diagnosis. The experimental study verified that this realized sensor has very small frequency shifts, significantly small electrical dimension and miniaturization, high sensitivity, and good linearity. The suggested configurations showed a capacity for sensing cancer cells in the GHz regime.

## 1. Introduction

Cancer remains one of the leading causes of death worldwide, underscoring the need for innovative methods to detect and identify this disease promptly. Direct approaches to cancer screening often rely on invasive processes or imaging methods with imperfect sensitivity and specificity. In recent years, there has been an increasing interest in taking advantage of advances in electromagnetic wave technology for accurate and non-invasive cancer detection in the THz regime [1,2,3,4,5]. There are other uses of metamaterial sensors in industry, such as fuel and oil adulteration detection [6], and in improving fifth-generation communication technology [7]. Metamaterials can exhibit strong field localization and amplification, allowing them to enhance sensor selectivity for detecting nonlinear substances and enable the detection of very small amounts upon analyses [8,9,10,11]. Based on this property, many new applications of metamaterials have recently been proposed. Metamaterial-based sensors offer significantly higher sensitivity compared to traditional sensors [12,13,14,15,16,17]. Similarly, the properties of metamaterial and fractal forms can be combined to obtain multi-service applications [18,19,20,21,22,23].

This study proposes a new biosensor to detect cancer cells using a corona-shaped metamaterial resonator. The use of metamaterials in the design of resonators has received considerable attention due to their unique electromagnetic properties and versatile functions. By combining the advantages of corona geometry and metamaterial structure, we aim to develop a resonance system capable of detecting cancer cells with high sensitivity and specificity. The resonator capacitance is proposed as the key factor enabling the detection of various cancer markers. Different types of cancer cells can exhibit distinct electromagnetic phenomena. By operating across multiple frequency bands, our resonator can efficiently capture and analyze these diverse signals, improving the accuracy and reliability of cancer detection. In addition, the coronal nature of the resonator allows for an increased surface area and better interaction of the electromagnetic wave, thereby increasing its sensing capabilities. Complete simulations, tests, and optimization studies were performed to confirm the proposed system’s performance. We simulated and measured the interaction between the resonator and cancer cells. By examining common signals, we could quantify the resonator’s overall sensitivity, specificity, and accuracy in detecting cancer cells.

The implications of this study are significant. By enabling immediate and non-invasive cancer detection, the proposed detection method promises to deliver compelling products to patients through early identification and rapid intervention. The development of advanced detection technologies in the field of oncology opens up new possibilities for more effective cancer management strategies, including personalized treatment planning and the monitoring of treatment effectiveness. This study aims to use the corona metamaterial resonator design to detect cancer cells sensitively and precisely. By pushing the boundaries of electromagnetic wave technology, we hope to contribute to ongoing efforts to revolutionize cancer diagnosis and improve patient care.

As with most research or studies, the goal is to try to eliminate the disadvantages and limitations of existing techniques to contribute to development and progress. The proposed biosensor is the result of a combination of the unique properties of metamaterials and corona-shaped geometry, which allows for increased surface area and better interaction of electromagnetic waves. This biosensor is designed to detect cancer cells experimentally, and distinguish them from healthy cells.

Two types of samples were used:

1. Samples of cancer cells circulating in the blood or specific tumor markers present in biological serum.
2. Tissue samples intended for histological studies.

The results will be presented in the following section. In both cases, there was a shift or variation in the resonance frequency, which explains the proper functioning of our biosensor.

Two different simulation approaches are used, leveraging the competences of two software platforms. The first method uses CST Studio Suite, which uses the finite element method (FEM) to perform the simulations. In contrast, the second approach makes use of ADS simulation software, which operates on the equivalent circuit method. This two-method approach allows a comprehensive exploration of the topic, and provides valuable insights from FEM-based simulations and those based on equivalent circuit methods. These versatile tools allow a more comprehensive analysis of the system under investigation, ensuring a comprehensive assessment of its performance, characteristics, and behavior. By harnessing the strengths of each simulation method, this study achieves a comprehensive and robust evaluation, which enriches understanding of the topic and enhances the reliability of the results obtained. Researchers can thus take advantage of the advantages of both FEM and equivalent circuit-based simulations, ensuring a more comprehensive perspective and deeper understanding of the phenomena under study. This integrated approach is essential to advance our knowledge in this field and make informed decisions based on a comprehensive evaluation of data and results.

Finally, the characteristics of this biosensor are compared with other resonators existing in the literature [24,25,26,27,28]. Most of this research only covers the simulation aspect without considering the verification of application performance [10,11,12,13,14,15,16,17,18,19,20,21,22,23,24,25,26,27,28], and this is what we present in this study, in a manner that applies across multiple samples.

## 2. Simulation Study of the Original Corona-Shaped Corona Metamaterial Biosensor for Cancer Cell Detection

**Figure 1:**
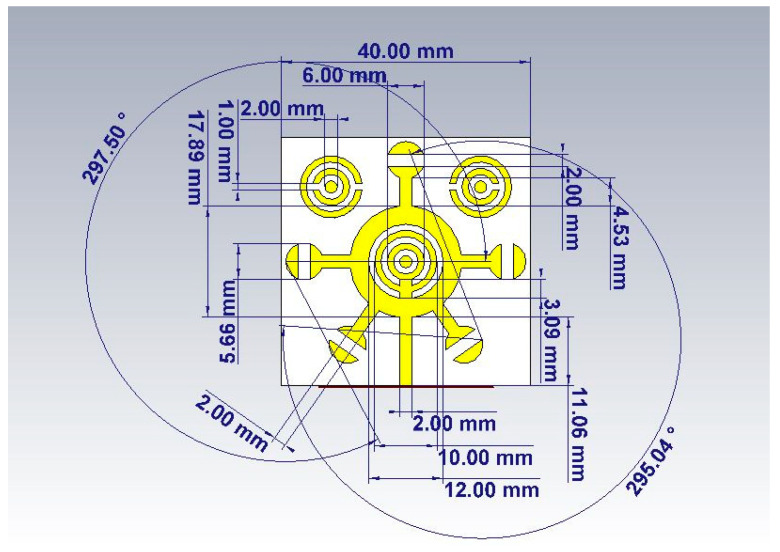
An illustration depicting the corona metamaterial resonator.

Before approaching the application aspect, which is considered very sensitive and vital to confirm the significance of this research, the quality and efficiency of the proposed biosensor are verified via simulation (see Figure 1). Figure 1 shows all the dimensions of this new design that were relied upon during the fabrication stage. The basic structure is a classic circle. The dimensions of the circular shape were calculated from the basic equations of transmission lines. The circular corona ring-shaped metamaterial was combined with a microstrip feed 2 mm in length and 2 mm in width. Two small circular ring-shaped metamaterial absorbers were added to the sides. A defected ground plane has a length of 40 mm and a width of 40 mm. The different optimal geometries’ dimensions and miniaturization sizes are shown in Figure 1.

**Figure 2:**
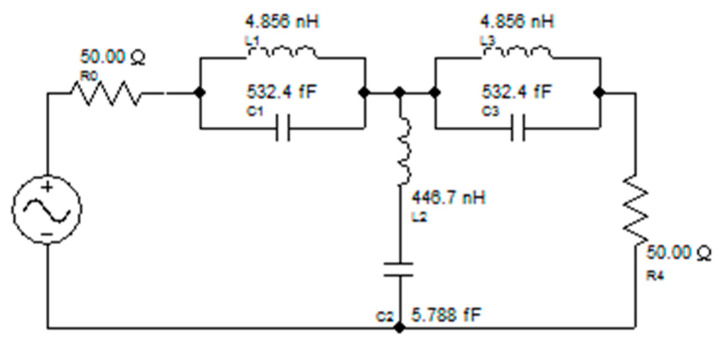
Equivalent circuit model of the corona metamaterial resonator.

**Figure 3:**
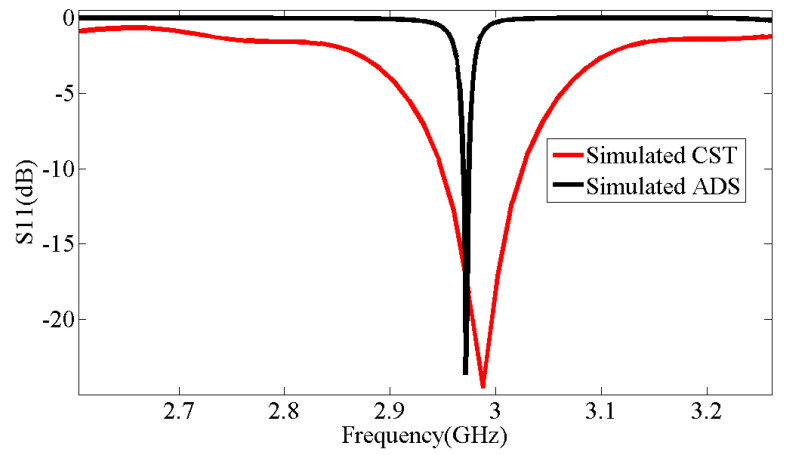
Comparison between simulated results of CST and simulated results of ADS.

An equivalent circuit is offered for the designed corona-shaped metamaterial biosensor, as shown in Figure 2. The equivalent circuit was extracted from CST software, which includes a feature for identifying the equivalent circuit for each model designed using it. This equivalent circuit was then designed using ADS software to ensure consistency in the results. Two different simulation approaches’ results are used (see Figure 3); one approach is simulation software CST Studio Suite based on the finite element method (FEM) (see Figure 1), and the other is simulation software ADS based on the equivalent circuit method, as shown in Figure 2. This ensures the convergence of the simulated results with the measured results is more reliable. Figure 3 shows a convergence between the results according to the two methods, where the resonance frequency obtained from the finite element method (FEM) is Fr = 2.988 GHz, and that obtained from the equivalent circuit method is Fr = 2.971 GHz.

**Figure 4:**
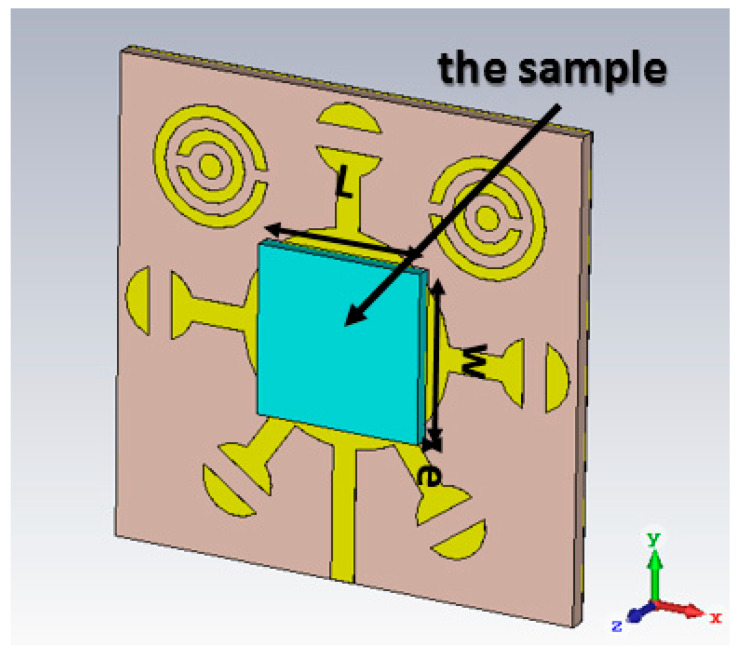
Three-dimensional view of the novel corona-shaped metamaterial biosensor with samples of thickness e = 2 mm, W = 7 mm and L = 7 mm.

**Figure 5:**
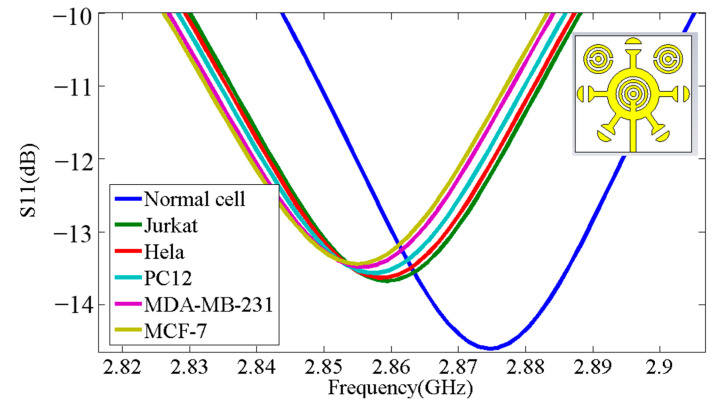
Simulation results of the S_11_ parameter response for different cancer cells.

**Figure 6:**
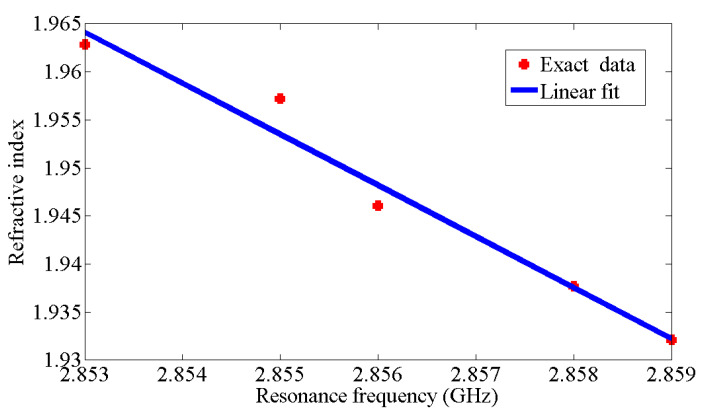
Relationship between the resonance frequency and refractive index (n) of cancer cells.

These properties enable the biosensor to effectively differentiate many different types of cancer cells, including basal cell; breast and cervical; Jurkat; MCF-7; and PC12 (see Figure 4). The resonant frequency and maximum attenuation of the biosensor (S11 dB) show remarkable sensitivity to changes in the sample’s refractive index, thereby achieving excellent linear performance, which is confirmed by the results presented in Table 1, where the index in the table was used from sources [10,11]. Figure 5 shows a direct correlation between the sample’s refractive index and varying resonance frequencies. Figure 6 shows the linear relationship between the resonance frequency and refractive index (n) of cancer cells.

The relationship between the resonance frequency and refractive index shows good linearity when using the following simple linear equation for the fitting procedure:

(1) $$n=-5.313f_{r}+17.12$$

The calculated sensitivity value can be expressed as follows:

(2) $$S=\frac{\Delta f_{r}}{\Delta n}=0.1825\;GHz/RIU$$

where the linear correlation R^2^ = 0.9696.

This biosensor has a margin of error of 0.647%.

**Figure 7:**
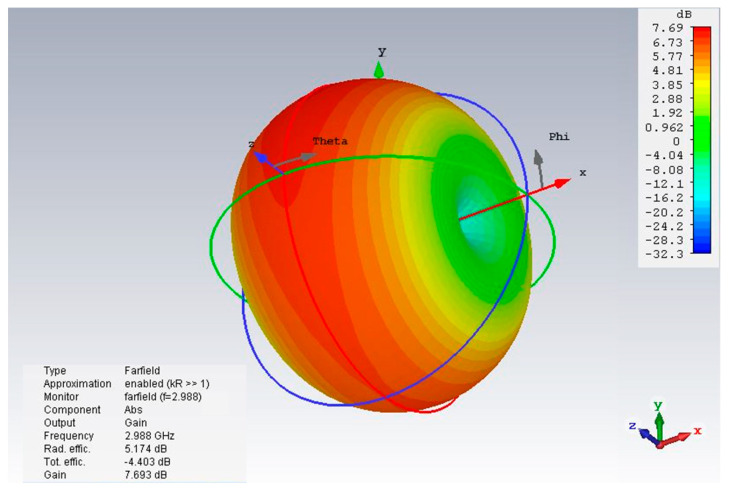
Three-dimensional radiation pattern of gain illustrated for the novel corona-shaped metamaterial biosensor operating at 2.988 GHz.

Figure 7 shows the high gain value of 7.69 dB for the novel corona-shaped metamaterial biosensor operating at 2.988 GHz. After presenting the simulation results, we concluded that this biosensor is designed to enable the detection of various cancer markers with high sensitivity, selectivity, and linearity properties. This allowed us to move to the next step, which is to compare the simulation results with the practical results and then verify its effectiveness in the experiment. These properties permit the biosensor to effectively differentiate many different types of cancer cells, including basal cell; breast and cervical; Jurkat; MCF-7; and PC12. The resonant frequency and maximum attenuation of the biosensor (S11 dB) showed significant sensitivity to changes in the refractive index of the sample, thereby achieving excellent linear performance.

## 3. Fabrication of the New Corona-Shaped Metamaterial Biosensor

**Figure 8:**
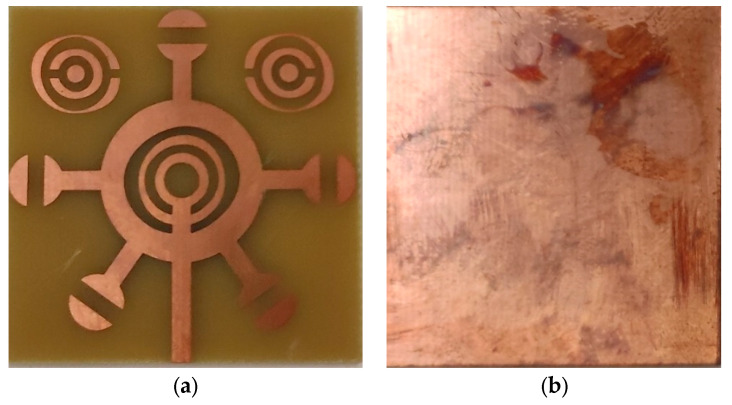
Photographs of corona-shaped metamaterial biosensor fabrication: (**a**) resonator corona-shaped metamaterial side, (**b**) side of ground plane.

This resonator is constructed on a dielectric substrate called FR4, which has a thickness of 1.56 mm (see Figure 8). The relative permittivity of FR4 is 4.3, and it has a loss tangent of 0.02. The metal pattern employed for the resonators is made of copper with a thickness of 0.035 mm. The copper used in the pattern has a conductivity of σ = 5.8 × 10^7^ S/m. In addition, the loading impedance of this microstrip line is 50 Ω.

**Figure 9:**
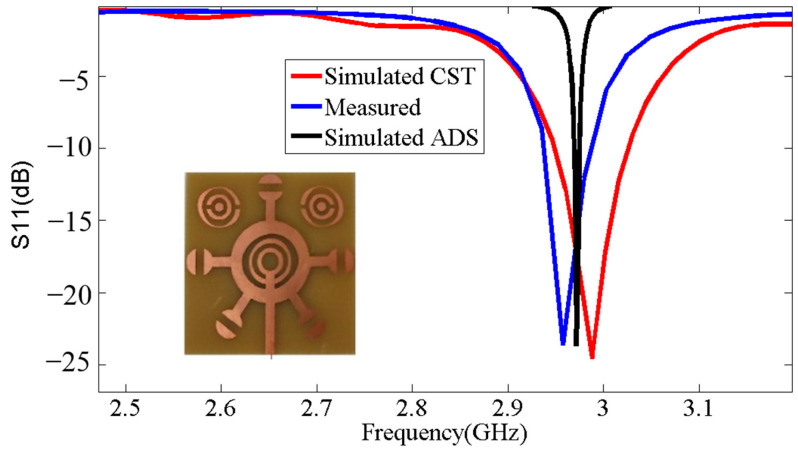
Comparison between simulated results and measured results.

Figure 9 displays the S11 curve simulated by CST, the S11 curve simulated by ADS, and the S11 curve of the corona metamaterial resonator measured using a network analyzer. Upon examining the two curves, it is evident that the practical results and the simulated results are nearly identical. Table 2 shows the resonance frequencies obtained from the simulations in CST, where Fr = 2.988 GHz; in ADS, where Fr = 2.971 GHz; and in practical observations, where Fr = 2.958 GHz. This correspondence confirms the manufacturing quality and its effectiveness in performing its function as a biosensor.

## 4. Experimental Characterization of the Corona Biosensor for Cancer Cell Detection

**Figure 10:**
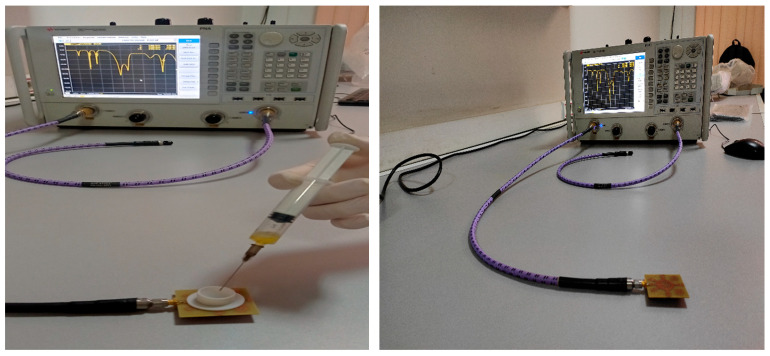
Resonator connected to the network analyzer for testing cancer cells.

Microwave sensors offer some benefits over conventional sensors. We particularly note their rapidity of measurements, accuracy, possibility of fully automation, and simplicity of production. In addition, non-destructive measurements can be made using microwave sensors. There are two types of microwave sensors: resonant and non-resonant. The advantages of resonance sensors are high sensitivity, stable signal, and low cost. The resonance frequencies and S11 parameters are analyzed to differentiate between negative tumor serum (non-cancerous) and positive tumor serum (cancerous). This research utilizes a novel corona metamaterial resonator (see Figure 10), which is a specially designed resonator that exhibits corona geometry. By exposing tumor serums to this biosensor, we aim to identify any variations in resonance frequencies and S11 parameters, which are indicators of electromagnetic comportment. By comparing the resonance frequencies and S11 parameters of negative tumor serum and positive tumor serum, this study aims to establish potential differences in the electromagnetic properties of these serums. This could potentially provide insights into the presence or absence of cancerous cells.

**Figure 11:**
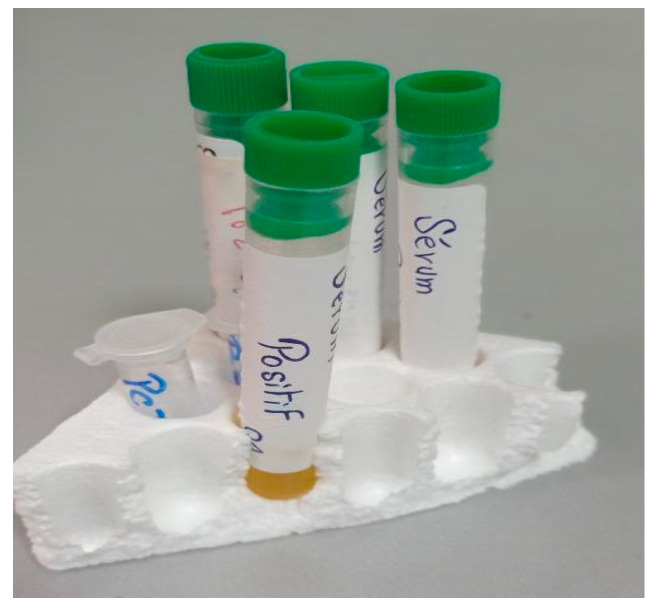
Samples obtained through circulating tumor cell detection.

In the first step (see Figure 11), the samples obtained through circulating tumor cell detection (CTC) are used. This method aims to detect and isolate cancer cells that have detached from a solid tumor and are circulating in the bloodstream. It is based on the principle that cancer cells release specific markers into the blood, such as antigens or tumor DNA. Techniques such as immunostaining, flow cytometry or PCR can be employed to detect and analyze these circulating tumor cells.

The negative serum contains the following (see Table 3).

**Table 3:** Composition of negative serum.

Parameters	Results	Norms
CA125 (Cancer Antigen 125)	4.50 U/mL	0.00–35.0
CA15-3 (Cancer Antigen 15-3)	3.98 U/mL	0.00–34.5
CA19-9 (Cancer Antigen 19-9)	4.11 U/mL	0.00–39.0

The positive serum 1 contains the following (see Table 4).

**Table 4:** Composition of positive serum 1.

Parameters	Results	Norms
CA125 (Cancer Antigen 125)	80.33 U/mL	0.00–35.0
CA19-9 (Cancer Antigen 19-9)	75.52 U/mL	0.00–39.0

**Figure 12:**
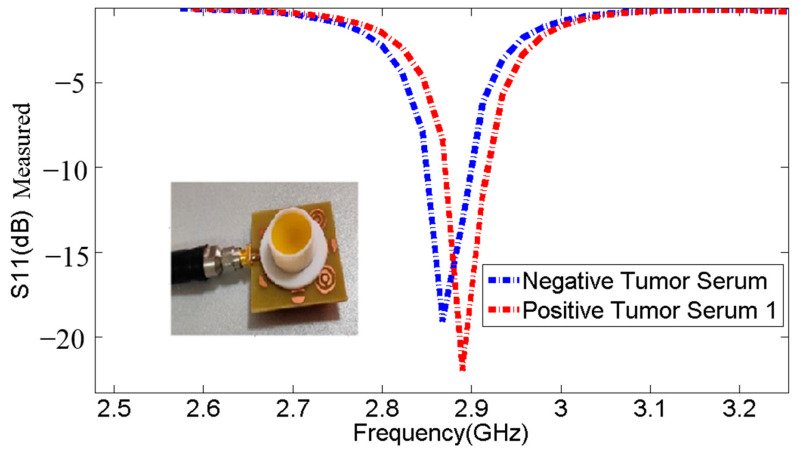
Comparison of resonance frequencies and S11 parameters between negative tumor serum and positive tumor serum 1.

**Figure 13:**
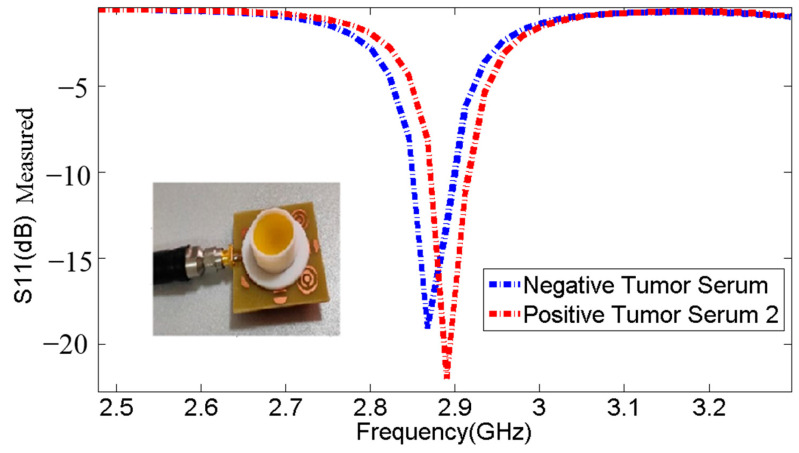
Comparison of resonance frequencies and S11 parameters between negative tumor serum and positive tumor serum 2.

**Figure 14:**
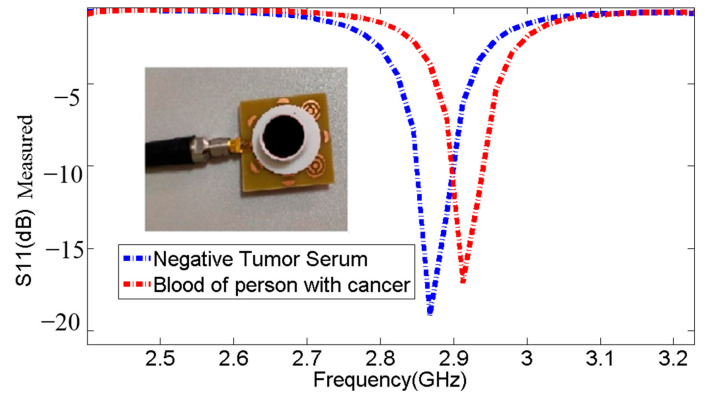
Comparison of resonance frequencies and S11 parameters between negative tumor serum and positive tumor marker results in patient blood.

The measurements were conducted to evaluate the biosensor’s ability to detect positive tumor serum. The findings in undisclosed Tables: Table 5, Table 6, Table 7, Table 8 and Table 9 and Figure 12, Figure 13 and Figure 14 demonstrate that the resonant frequency increases when detecting positive tumor serum. Additionally, the resonant frequency for positive tumor serum is observed at 2.89 GHz, while for negative tumor serum, it is observed at 2.868 GHz, representing a shift of 22 MHz. These observations indicate a proportional relationship when comparing the resonance frequencies and S11 parameters of negative tumor serum and positive tumor serum.

The positive serum 2 contains the following (see Table 6).

**Table 6:** Composition of positive serum 2.

Parameters	Results	Norms
CA15-3 (Cancer Antigen 15-3)	>300 U/mL	0.00–34.5

**Table 7:** Comparison of resonance frequencies and S11 parameters between negative tumor serum and positive tumor serum 2.

Sample	Frequencies (GHz)	S11 (dB)
**Without Sample**	2.958	−23.63
Negative Tumor Serum	2.868	−19.08
Positive Tumor Serum	2.89	−21.97

The blood of a person with breast cancer contained the following (see Table 8).

**Table 8:** Positive tumor marker results in the patient’s blood.

Parameters	Results	Norms
CA15-3 (Cancer Antigen 15-3)	42.20 U/mL	0.00–34.5

**Table 9:** Comparison of resonance frequencies and S11 parameters between negative tumor serum and positive tumor marker results in patient blood.

Sample	Frequencies (GHz)	S11 (dB)
**Without Sample**	2.958	−23.63
Negative Tumor Serum	2.868	−19.08
Blood of a person with cancer	2.913	−17.8

PreciControl Tumor Marker Level 2 refers to a specific type of tumor marker testing or assay. Tumor markers are substances found in the blood, urine, or tissues of individuals with certain types of cancer. They are often used in cancer diagnosis, monitoring treatment response, and detecting cancer recurrence.

PreciControl is likely the name of a specific tumor marker test or assay, and “Tumor Marker Level 2” indicates a particular level or category of the tumor marker result.

**Figure 15:**
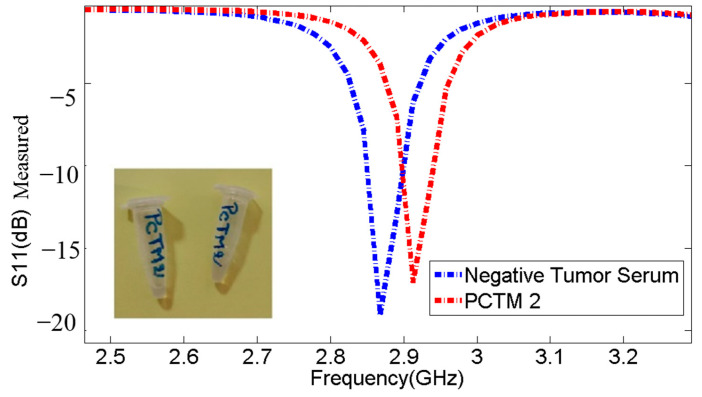
Comparison of resonance frequencies and S11 parameters between negative tumor serum and PCTM 2.

The marks offered in undisclosed Table 10 and Figure 15 validate that the resonant frequencies increase, respectively, when detecting PCTM 2. In addition, the resonant frequency for PCTM 2 is observed at 2.913 GHz, with S11 of −17.09 dB, while the negative tumor serum is observed at 2.868 GHz, with S11 of −19.08, representing a frequency shift of 45 MHz.

**Figure 16:**
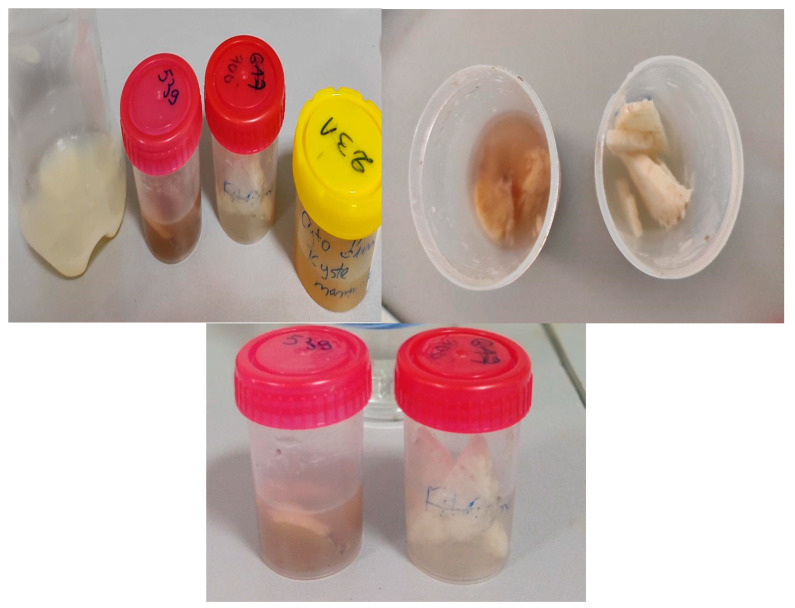
Biopsy samples.

In the second step (see Figure 16), we collected samples by performing biopsies using the histopathological examination of tissues. This method involves taking a tissue sample from the suspected area and examining it under a microscope to detect the presence of cancer cells.

**Figure 17:**
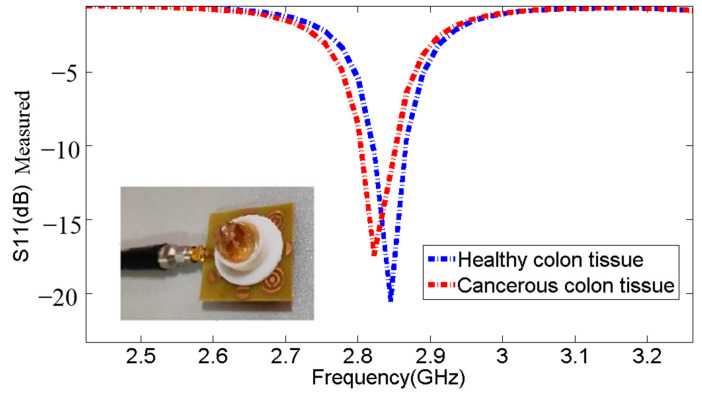
Comparison of biopsy samples—healthy colon vs. cancerous colon.

Figure 17 compares two biopsy samples: one from a healthy colon and the other from a colon affected by cancer. The displayed images or data highlight the distinct differences between the healthy tissue and the cancerous tissue, providing visual evidence of our sensor’s detection capabilities and proper functioning in distinguishing between the two types.

The undisclosed Table 11 and Figure 17 results demonstrated that the resonant frequencies decrease when detecting cancerous colon cells. Additionally, the resonant frequency for cancerous colon cells is observed at 2.845 GHz, with S11 of −17.45 dB, while the resonant frequency for healthy colon cells is observed at 2.822 GHz, with S11 of −20.56 dB, representing a frequency shift of 23 MHz.

This research investigated the use of a unique corona metamaterial biosensor to distinguish between negative and positive tumor serums based on their electromagnetic characteristics. The findings could have implications for future non-invasive cancer diagnostic techniques. We have validated the key performance features of our biosensor by conducting a thorough comparison with similar devices (Table 12). By assessing its essential characteristics, we have substantiated the effectiveness and significance of our biosensor compared to the state of existing and recent research [24,25,26,27,28]. This study proves its superior performance and relevance within the current landscape. This experimental research has demonstrated that this biosensor has very small frequency variation, a significantly smaller size and electrical miniaturization, high sensitivity, and good linearity. The proposed structures show the ability to detect cancer cells.

## 5. Conclusions

Developing a novel corona metamaterial biosensor for cell cancer detection holds promise for improving cancer detection and diagnosis. By exploiting the unique properties of corona geometry and metamaterial structure, this resonator increases sensitivity and specificity in capturing electromagnetic signals associated with other cancer markers. This capability enables the detection of a wide variety of cancers, featuring a halo to maximize surface area and enhance electromagnetic wave interaction. Through rigorous simulation and optimization studies, the efficiency of the resonance system is proposed. The non-invasive and real-time nature of this detection technology shows the potential for development into cancer surveillance strategies, enabling rapid research and verification of treatment efficacy. Further advances in this area could lead to better patient outcomes and a paradigm shift in approaches to cancer detection and impact. Overall, the halo metamaterial resonator represents a promising avenue for improving cancer detection and contributing to the fight against this devastating disease. This paper on corona geometry metamaterial structures holds promise beyond cancer detection, potentially benefiting diverse medical fields, including advanced studies on viruses like COVID-19 (SARS-CoV-2).

## Figures and Tables

**Table 1:** The S_11_ parameter response of samples.

Cancer Cell	Refractive Index	|S_11_| dB	Resonant Frequency (GHz)	Refractive Index from Fitting	Error Δn/n (%)
Normal cell	1.8225	14.6	2.874	1.8505	1.536
Jurkat	1.932100	13.67	2.859	1.930133	0.102
Hela	1.937660	13.63	2.858	1.93545	0.114
PC12	1.946025	13.56	2.856	1.9560	0.81
MDA-MB-231	1.957201	13.48	2.855	1.9514	0.58
MCF-7	1.962801	13.43	2.853	1.97201	0.469

**Table 2:** Comparison of resonance frequencies and S11 parameters between simulated and measured values.

Parameter	Frequencies (GHz)	S11 (dB)
Simulated by CST	2.988	−24.55
Simulated by ADS	2.971	−23.67
Measured	2.958	−23.63

**Table 5:** Comparison of resonance frequencies and S11 parameters between negative tumor serum and positive tumor serum.

Sample	Frequencies (GHz)	S11 (dB)
**Without Sample**	2.958	−23.63
Negative Tumor Serum	2.868	−19.08
Positive Tumor Serum	2.89	−21.97

**Table 10:** Comparison of resonance frequencies and S11 parameters between negative tumor serum and PCTM 2.

Sample	Frequencies (GHz)	S11 (dB)
**Without Sample**	2.958	−23.63
Negative Tumor Serum	2.868	−19.08
PCTM 2	2.913	−17.09

**Table 11:** Comparison of resonance frequencies and S11 parameters between biopsy samples—healthy colon vs. cancerous colon.

Sample	Frequencies (GHz)	S11 (dB)
**Without Sample**	2.958	−23.63
A specimen of healthy colon tissue	2.845	−20.56
A specimen of cancerous colon tissue	2.822	−17.45

**Table 12:** Comparison of sensing performance with existing sensors.

Reference Year	Resonator Design Size	Return Loss [S11] dB	Gain dB	Radiation Pattern
2016 [24]	$65\;\times \;50\;{mm}^{2}$	−42.5	1.01	Bidirectional
2018 [25]	$46.4\;\times \;36.8\;{mm}^{2}$	<−10	12.6	Directional
2020 [26]	Overall dimension 3670.4 mm^3^	<10	2.9	Bidirectional
2021 [27]	$60\;\times \;19\;{mm}^{2}$	−17.125	1.508	Directional
2022 [28]	$70\;\times \;70\;{mm}^{2}$	−11.68	4.02	Unidirectional
This work	$40\;\times \;40\;{mm}^{2}$	−23.63/−24.55	7.69	Directional

## Data Availability

No new data were created in this study. Data sharing is not applicable to this article.
